# Erythropoietin as an add-on treatment for cognitive side effects of electroconvulsive therapy: a study protocol for a randomized controlled trial

**DOI:** 10.1186/s13063-018-2627-2

**Published:** 2018-04-19

**Authors:** Lejla Sjanic Schmidt, Jeff Zarp Petersen, Maj Vinberg, Ida Hageman, Niels Vidiendal Olsen, Lars Vedel Kessing, Martin Balslev Jørgensen, Kamilla Woznica Miskowiak

**Affiliations:** 1 Copenhagen Affective Disorder Research Center (CADIC), Psychiatric Center Copenhagen, Copenhagen University Hospital, Rigshospitalet, Copenhagen, Denmark; 2 Department of Psychology, University of Copenhagen, Øster Farimagsgade 2A, DK-1353 Copenhagen, Denmark; 3 Faculty of Health and Medical Sciences, University of Copenhagen, Copenhagen, Denmark; 4 Department of Biomedical Sciences, University of Copenhagen, Copenhagen, Denmark; 5 Department of Neuroanaesthesia, The Neuroscience Centre, Copenhagen University Hospital, Rigshospitalet, Copenhagen, Denmark; 6 Neurocognition and Emotion in Affective Disorder (NEAD) Group, Copenhagen Affective Disorder Research Center, Psychiatric Center Copenhagen, Copenhagen University Hospital, Rigshospitalet, Blegdamsvej 9, DK-2100 Copenhagen, Denmark

**Keywords:** Depression, Unipolar disorder, Bipolar disorder, Electroconvulsive therapy, Cognition, Cognitive side effects, Erythropoietin, Functional magnetic resonance imaging

## Abstract

**Background:**

Electroconvulsive therapy (ECT) is the most effective treatment for severe depression, but its use is impeded by its cognitive side effects. Novel treatments that can counteract these side effects may therefore improve current treatment strategies for depression. The present randomized trial investigates (1) whether short-term add-on treatment with erythropoietin (EPO) can reduce the cognitive side -effects of ECT and (2) whether such effects are long-lasting. Further, structural and functional magnetic resonance imaging (MRI) will be used to explore the neural underpinnings of such beneficial effects of EPO. Finally, the trial examines whether potential protective effects of EPO on cognition are accompanied by changes in markers of oxidative stress, inflammation, and neuroplasticity.

**Methods/design:**

The trial has a double-blind, randomized, placebo-controlled, parallel group design. Patients with unipolar or bipolar disorder with current moderate to severe depression referred to ECT (*N* = 52) are randomized to receive four high-dose infusions of EPO (40,000 IU/ml) or placebo (saline). The first EPO/saline infusion is administered within 24 h before the first ECT. The following three infusions are administered at weekly intervals immediately after ECT sessions 1, 4, and 7. Cognition assessments are conducted at baseline, after the final EPO/saline infusion (3 days after eight ECT sessions), and at a 3 months follow-up after ECT treatment completion. The neuronal substrates for potential cognitive benefits of EPO are investigated with structural and functional MRI after the final EPO/saline infusion. The primary outcome is change from baseline to after EPO treatment (3 days after eight ECT sessions) in a cognitive composite score spanning attention, psychomotor speed, and executive functions. With a sample size of *N* = 52 (*n* = 26 per group), we have ≥ 80% power to detect a clinically relevant between-group difference in the primary outcome measure at an alpha level of 5% (two-sided test). Behavioral, mood, and blood-biomarker data will be analyzed using repeated measures analysis of covariance. Functional MRI data will be preprocessed and analyzed using the FMRIB Software Library.

**Discussion:**

If EPO is found to reduce the cognitive side effects of ECT, this could have important implications for future treatment strategies for depression and for the scientific understanding of the neurobiological etiology of cognitive dysfunction in patients treated with ECT.

**Trial registration:**

ClinicalTrials.gov, NCT03339596. Registered on 10 November 2017.

**Electronic supplementary material:**

The online version of this article (10.1186/s13063-018-2627-2) contains supplementary material, which is available to authorized users.

## Background

Electroconvulsive therapy (ECT) is an effective, fast-acting, and safe treatment for severe depression [1], but it is associated with troublesome cognitive side effects across episodic memory, attention, and executive functions that persist for weeks [2, 3] to months after treatment completion [4, 5]. Such cognitive side effects of ECT are the greatest impediment to its prescription for depression, making it reserved for only the most severely ill patients [6]. Therefore, novel treatments that counteract these side effects have the potential to improve current treatment strategies by allowing a greater number of severely ill patients to get earlier and more effective treatment with fewer cognitive side effects. However, research efforts into identification of methods to attenuate the cognitive side effects of ECT treatment without hampering its clinical efficacy have thus far been unsuccessful [7].

Erythropoietin (EPO) is a promising treatment for cognitive dysfunction in mood disorders [8, 9] and for counteracting ECT-induced cognitive side effects. EPO is not only produced systematically in the kidneys, but is also produced in the brain, where it mediates neuroprotection and development, modulates oxidative stress and inflammation [10], and plays a key role in cognitive functioning [11, 12]. Systemically administered EPO crosses the blood-brain barrier and has been shown in preclinical studies to mediate neuroprotection and neuroplasticity and to enhance cognitive functions when given in high doses (≥ 500 IU/kg body weight) [13, 14]. Randomized controlled clinical studies suggest that 8–12 weeks of systemically administered high-dose (40,000–48,000 IU) EPO improves attention, memory, and executive functions in patients with treatment-resistant depression (TRD) [9], bipolar disorder (BD) [8], multiple sclerosis [15], or schizophrenia [16]. The cognitive benefits of EPO treatment seem to result from direct neurobiological actions rather than non-specific changes in red blood cells. For example, randomized placebo-controlled functional magnetic resonance imaging (fMRI) studies by our group [17, 18] showed that a single high dose (40,000 IU) of EPO vs. placebo enhanced memory-relevant prefrontal and hippocampal activity in healthy and depressed individuals without affecting red blood cells. Consistent with this, our subsequent randomized, placebo-controlled trials revealed that eight weekly infusions of high-dose (40,000 IU) EPO vs. saline had mood-independent beneficial effects on cognitive function in patients with TRD (*N* = 40) and BD in remission (*N* = 44) [8, 9]. These cognitive benefits were accompanied by EPO-associated increase in neural activity within the frontal and the parietal lobes during strategic encoding and working memory tests [8, 9, 19]. Notably, EPO-related memory improvement in these patients was associated with reversal of hippocampal (cornu ammonis 1–3) and subiculum volume loss [20], which is interesting in light of ECT induction of hippocampal volume increase (see, e.g., [21]). Importantly, these brain changes were independent of changes in mood and lasted long term beyond red blood cell normalization. Several neurobiological actions may underlie these beneficial cognitive effects of EPO treatment, including activation of anti-inflammatory, anti-apoptotic, and antioxidant signaling pathways [14, 22, 23] and growth of dendrites, maturation of neural progenitor cells, and upregulation of brain-derived neurotrophic factor (BDNF) [24, 25]. Taken together, preliminary findings highlight EPO as a candidate treatment for ECT-induced cognitive deficits in mood disorders. This trial extends our previous work by investigating for the first time whether adjunctive EPO treatment can counteract the cognitive side effects of ECT.

### Aims and hypotheses

The present trial aims to investigate whether one primer infusion of EPO/saline before ECT followed by three weekly EPO/saline infusions during the course of ECT counteracts cognitive side effects in patients receiving ECT. Furthermore, we aim to investigate the neuronal underpinnings of such potential effects of EPO with structural and functional magnetic resonance imaging (MRI) and the role of oxidative, inflammatory, and neuroendocrinological systems with blood and urine tests.

We hypothesize that EPO treatment will (1) counteract ECT-induced decline in cognition (primary endpoint), which will be accompanied by (2) increased subregional hippocampal volume, (3) greater memory-related hippocampal activation and reinforcement of dorsolateral prefrontal activity during memory encoding and working memory, and (4) changes in peripheral markers of inflammation, oxidative stress, and neuroplasticity. Given preliminary evidence for the beneficial effects of EPO on depression-relevant outcomes [8, 13], a secondary hypothesis is that add-on EPO treatment will produce greater, more sustained mood improvement than ECT treatment alone.

## Methods/design

### Participants and screening

A total of 52 patients with major depression (MDD) or BD scheduled for ECT treatment will be recruited from Psychiatric Centers in The Mental Health Services in the Capital Region of Denmark. Half of the participants will be randomized to receive active EPO treatment (*n* = 26), while the other half will receive a placebo (saline) (*n* = 26).

Prior to enrollment, patients will be screened with the *Mini International Neuropsychiatric Interview* (MINI) [26] to confirm their International Statistical Classification of Diseases and Related Health Problems (ICD-10) diagnosis. Eligible patients have a diagnosis of MDD, unipolar disorder (UD), or BD with current moderate to severe depressive episode symptoms, a Hamilton Depression Rating Scale, 17 items (HDRS-17) score ≥ 17 [27], are 18–70 years of age, have fluent Danish skills, and are able to provide informed consent. Exclusion criteria are treatment under involuntary measures, previous ECT within the last 3 months, other neuropsychiatric conditions, alcohol or substance misuse disorder, or recent suicide attempts. To ensure the safety of the EPO treatment, patients are also excluded if they have a significant medical condition (including diabetes, renal failure, heart disease, epilepsy, untreated/insufficiently treated hypertension, malignancies, or thromboses), are pregnant, use contraceptive medication, or have a family history of thromboses or epilepsy, similar to our previous studies [28]. Pregnancy tests are mandatory for and will be performed on female patients in their fertile age before their inclusion in the trial. Patients will also be excluded if they are overweight (body mass index (BMI) > 30) or have a body weight < 45/> 95 kg. Blood screening and physical examinations are undertaken at baseline and weekly during the 3-week EPO treatment period to ensure patient safety. These exclusion criteria and weekly safety monitoring prevented serious adverse events in our previous EPO trials [8, 9, 28]. Written informed consent is obtained by one of the named authors before inclusion. The procedures are in accordance with the ethical standards of the Danish Research Ethics Committee for the Capital Region.

### Setting

Patients will be randomized to receive a total of four intravenous infusions of either recombinant human EPO (40,000 IU/ml; Epoetin alfa; Eprex, Janssen-Cilag) or a placebo (1 ml NaCl) diluted with 100 ml saline (0.9% NaCl) that is administered over 15 min. The first infusion is given within 24 h before the first ECT, and the following three infusions are administered immediately after ECT at weekly intervals (after ECT sessions 1, 4, and 7). The treatment and all the outcome assessments will take place at Psychiatric Center Copenhagen, Psychiatric Center Glostrup, or Psychiatric Center Amager. Functional MRI is conducted at Copenhagen University Hospital, Rigshospitalet. Participants will be transported to and back from Copenhagen University Hospital in a safe setting by two members of our research team. Furthermore, to ensure the quality and the reliability of the blood test results, all the blood samples will be analyzed at the same laboratory, also at the Copenhagen University Hospital, Rigshospitalet.

### Study design and procedures

**Fig. 1:**
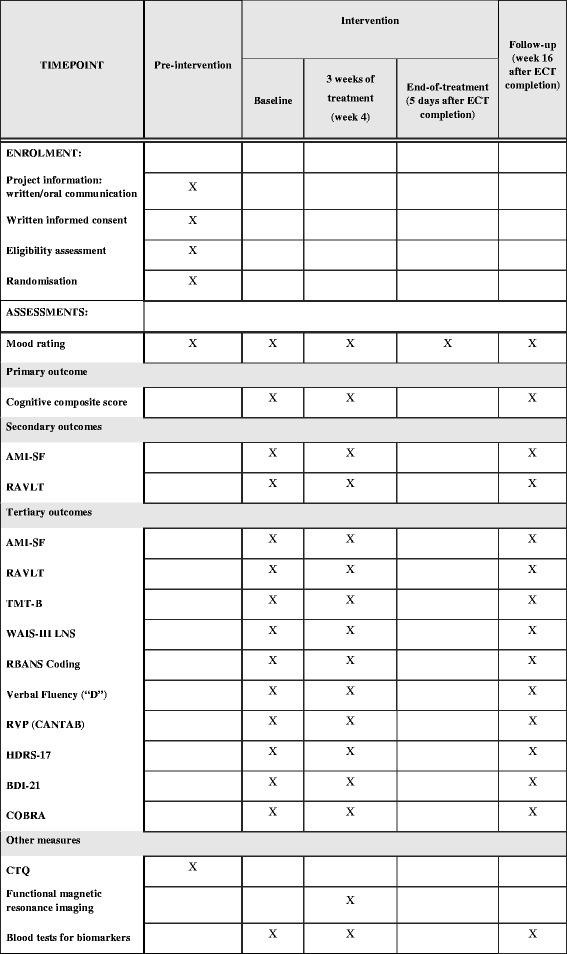
Schedule of enrollment, interventions, and assessments. Abbreviations: *AMI-SF* Columbia University Autobiographical Memory Interview-Short Form, *RAVLT* Rey Auditory Verbal Learning Test, *TMT-B* Trail Making Test Part B, *WAIS-III LNS* Wechsler Adult Intelligence Scale Version III Letter-Number Sequencing, *RBANS Coding* Repeatable Battery for the Assessment of Neuropsychological Status Coding, *RVP* Rapid Visual Processing (*CANTAB* Cambridge Cognition Ltd.), *HDRS-17* Hamilton Depression Rating Scale, 17-item version, *BDI-21* Beck Depression Inventory, 21 items, *COBRA* Cognitive Complaints in Bipolar Disorder Rating Assessment, *CTQ* Childhood Trauma Questionnaire

The trial has a randomized, double-blinded, placebo-controlled, parallel group design. The study design and procedures are summarized in Fig. 1. The Standard Protocol Items: Recommendations for Interventional Trials (SPIRIT) checklist is provided in Additional file 1. Cognitive functions, mood symptoms, and blood and urine markers of inflammation, oxidative stress, and neuroplasticity will be assessed three times during the trial. The first time will be at baseline, the second time post-EPO treatment 3 days after ECT session 8 (patients skip one ECT session day after eight ECTs to minimize the confounding effects of *acute* side effects of ECT due to anesthesia, etc.), and the third time at a 3 months follow-up after ECT completion. In addition, the neuronal substrates for potential effects of EPO on cognition are investigated with structural and fMRI after eight ECT sessions (i.e., after three weekly EPO or saline infusions). The rationale for assessing cognition and neuronal activity after eight ECTs is to ensure participation of almost all patients, given our experience that > 95% of patients need eight or more ECTs (unpublished observations from [29]) post-treatment. EPO/saline will be administered when patients wake up following ECT. The EPO doses are identical to those found to modulate neural and cognitive function with short-term administration [17] and to improve cognition with long-term treatment [8, 9, 16, 30]. Concomitant medication is kept stable for the duration of the study, unless the individual patient’s psychiatrist deems it necessary to change medication.

EPO is kept at 2–8 °C during transport and storage to minimize the risk of damaging the medication and potential side effects. EPO will be dissolved in 100 ml saline and administered intravenously over 15 min. Patients will stay at the clinic for observation for at least 30 min after each infusion in case of acute severe adverse events. In cases of significantly increased hematocrit (men > 50%, women > 48%) at two consecutive blood test measurements within the same week, bloodletting (450 ml) will be performed on a weekly basis with no cessation of treatment until hematocrit values are normalized. In the case of a significant increase in thrombocytes (> 400 billion/L) or a drop in reticulocytes (< 1‰), two repeated controls will be performed in the following week. If the values stay abnormal, the patient will be withdrawn from further study participation and monitored with weekly medical examinations and blood samples until he or she is stabilized. If deemed necessary by the medic responsible for patient safety, the patient might be hospitalized for observation. Procedures for breaking randomization codes are established in the case of serious adverse events potentially or directly related to the medical intervention. These procedures will be followed if knowledge of the patient’s medical treatment will have implications for treatment of the observed serious adverse events. The principal investigator or any medical doctor responsible for the patient is able to decide whether to break emergency envelopes for potentially affected patients.

### ECT procedures

ECT will be administered three times per week (Mondays, Wednesdays, and Fridays), according to the standard protocol of the Mental Health Services in the Capital Region of Denmark. Patients are anesthetized with thiopental, and succinylcholine is administered for muscle relaxation. Bitemporal (BT) electrode placement with an energy dosage 1.5 times above the seizure threshold can be changed to right unilateral (RUL) treatment in the case of severe cognitive side effects; this is decided by the treating psychiatrist. The dosing strategy with the initial dose is based on patient age (charge [percentage of 500 millicoulombs] = 50% of the age). Subsequent treatments are dose-titrated based on seizure quality. The end of treatment is determined by the treating psychiatrist.

### Randomization and blinding

Power calculation and block randomization have been conducted by the independent *Pharma Consulting Group AB* (www.pharmaconsultinggroup.com) using PROC POWER and the SAS code in the Statistical Analysis Software (SAS) version 3. Treatment groups are stratified for age (≥ 40 or < 40) and gender. At the time of inclusion, gender and date of birth are registered to determine the appropriate stratum for each included patient. Study identification numbers are provided consecutively within each stratum. Patients and outcome assessors are blinded to treatment assignment, and blinding is maintained throughout the study period and the data analysis. The randomization list is kept in a locked filing cabinet for which only the person preparing the study medication and the principal investigator have a key. Preparation of study medication is performed to ensure double-blinding at the time of infusion: 1 ml recombinant human EPO (Eprex; 40,000 IU; Janssen-Cilag) or saline (NaCl 0.9%) kept at a temperature of 5–8 °C is injected into a standard 100 ml saline (NaCl 0.9%) infusion bag, which is then given to the study nurse or physician administering the medication. Double-blinding is further ensured by EPO being a colorless liquid undistinguishable from saline. Weekly monitoring of blood tests and any side effects of EPO treatment are performed by a physician not involved in outcome measure assessments. Participants are instructed to not reveal any symptoms or potential physical side effects associated with EPO treatment to study personnel involved in outcome assessments. The Good Clinical Practice (GCP) Unit at Copenhagen University Hospital (www.gcp-enhed.dk/kbh) will monitor that blinding is maintained. Unblinding for individual participants is permitted for safety reasons in cases of side effects or serious adverse events likely or directly related to the study medication. Revealing a patient’s allocated intervention during the trial involves opening his or her sealed randomization envelope. In these cases, it is the sovereign decision of LSS, MBJ, MV, or LVK as to whether the randomization code should be broken.

### Outcome assessments

For an overview of frequency and timing of outcome assessment, see Fig. 1.

The *primary outcome* comprises change from baseline to post-treatment on a cognitive composite score assessing attention, verbal learning and memory, and executive functions. We have found an improvement on this “speed of complex cognitive processing” composite measure in our previous EPO cognition trial across patients with BD and TRD after 8 weeks of weekly EPO treatment [31]. In the present study, we therefore include the same global cognition score as the primary outcome measure. This consists of the following six neuropsychological tests, spanning verbal memory, attention, and executive functions: Rey Auditory Verbal Learning Test (RAVLT) [32, 33], the Repeatable Battery for the Assessment of Neuropsychological Status (RBANS) Coding [34], Verbal Fluency with the letter “D” [35], Wechsler Adult Intelligence Scale (WAIS)-III Letter-Number Sequencing [36], Trail Making Test Part B (TMT-B) [37], and Rapid Visual (Information) Processing (RVP) from the Cambridge Neuropsychological Test Automated Battery (CANTAB Cognition Ltd.). The secondary outcomes are retrograde autobiographical memory measured with the Columbia University Autobiographical Memory Interview-Short Form (AMI-SF) [38, 39] and verbal learning and memory assessed with the RAVLT [32, 33]. The tertiary outcomes comprise (1) individual neuropsychological tests of the cognitive composite score, (2) depression severity assessed with the HDRS-17 [27] and the Beck Depression Inventory, 21 items (BDI-21 [40]), respectively, (3) subjectively rated cognitive complaints assessed with the Cognitive Complaints in Bipolar Disorder Rating Assessment (COBRA) [41], (4) cognition-related activity in key neuronal circuits and hippocampal structure, and (5) blood- and urine-based markers of inflammation, oxidative stress, and neuroplasticity. History of early life stress will be assessed with the Childhood Trauma Questionnaire (CTQ [42]) at the eligibility assessment.

To reduce risks of learning effects at follow-up neuropsychological assessments, we administer alternate versions of the RAVLT (original list AB, GeAB, and Cr-AB) and RBANS Coding (version A and B) [34] in a counterbalanced order within each stratum.

### Peripheral and neural biomarkers and genotyping

To investigate whether there is a significant difference between EPO- and saline-treated patients in structural and functional measures of cognition-relevant neural regions, including the hippocampus and the frontal lobes, patients undergo a structural and fMRI scan after 3 weeks of weekly EPO or placebo infusions (i.e., at week 4). During fMRI (approximately 45 min duration) patients are given three neurocognitive tests: (1) the Autobiographical Memory Test [43] adapted for fMRI assessment [13, 44], (2) a hippocampus-dependent picture memory retrieval test that is sensitive to the effects of EPO on hippocampal response [17], and (3) a Spatial N-back working memory test from our previous study [19]. Furthermore, we will examine whether the potential protective effects of EPO on cognition are accompanied by changes in markers of oxidative stress, inflammation, or neuroplasticity in blood and urine. Specifically, blood samples will be analyzed to investigate whether changes in the peripheral biomarker, high-sensitivity C-reactive protein (hsCRP), are important for cognitive benefits of EPO treatment.

### Biochemistry

Since EPO has anti-inflammatory actions [14, 22, 23] and compiling evidence indicates that major depressive disorder (MDD) is associated with increased low-grade neuroinflammation [45–47], we will explore the effects of add-on treatment with EPO on systemic markers of inflammation. Blood and urine samples will be transferred to the Neuropsychiatric Laboratory, Department O, Rigshospitalet, and stored at − 80 °C until use. Measurements will be performed at the Neuropsychiatric Laboratory and at the Department of Clinical Pharmacology, Rigshospitalet.

### Statistical analyses

The statistical significance threshold is considered to be *p* < 0.05 (two-tailed). Behavioral, mood, and biomarker data will be analyzed using a mixed models design and an intention-to-treat (ITT) approach. Structural and functional imaging data will be collected using a 3 T MR scanner operating at Copenhagen University Hospital, Rigshospitalet. Resting state and task-related fMRI data will be preprocessed and analyzed using the FMRIB Expert Analysis Tool (FEAT) and the ‘randomize’ algorithm integrated in the FMRIB Software Library (FSL), www.fmrib.ox.ac.uk/fsl. We will adjust for potential differences in demographical and clinical parameters at baseline. All statistical analyses will be run using the *Statistical Package for Social Sciences* (SPSS, version 23, IBM Corporation, Armonk, NY).

#### Sample size and power calculation

The sample size and statistical power have been calculated by Pharma Consulting Group AB using PROC POWER and the SAS code in SAS version 3. We estimate that a clinically relevant differential change in the cognitive composite score between the EPO and placebo groups is 0.4 standard deviations (SD; corresponding to a moderate effect size), with an SD of the mean change of 0.5. This is consistent with the recommendations of the International Society for Bipolar Disorders (ISBD) cognition task force [48]. Specifically, the task force noted that a differential change between groups of 0.2–0.4 SD on a global composite score represents a potentially clinically relevant change, as this may translate into moderate to large functional improvement in patients with mood disorders [48]. In our previous 8-week EPO trial, the difference between EPO and the saline groups regarding change in the cognitive composite score from baseline to post-treatment was 0.5 SD [31]. Based on the ISBD task force recommendations and our previous findings regarding the effects of longer term EPO treatment, we estimate that a sample size of *N* = 52 (*n* = 26 per group) will reach a ≥ 0.8 power to detect a similar clinically relevant differential change of 0.4 SD in the primary outcome measure (the cognitive composite score) with an SD of this change of 0.5 between the two groups at an alpha level of 5% (two-sided test) [31].

The study is also powered to investigate differences in fMRI blood-oxygen-level dependent (BOLD) response in key neural networks based on our previous fMRI studies, in which sample sizes of 30 age- and gender-matched participants (*n* = 15 per group) had a power of > 0.8 to show drug-related effects on task-related neural response (e.g., memory and executive function) at an alpha level of *p* < 0.05 [19, 31]. In the current trial, the inclusion of 52 participants (*n* = 26 per treatment group) therefore ensures sufficient statistical power to detect EPO-related effects on neural activity.

### Data management and monitoring

Personal information is obtained at enrollment or from patient records, if patients are unable to provide the necessary information. Written informed consent forms will be signed and kept in a locked filing cabinet, and a password-protected list that matches participant ID numbers with personal information will be stored isolated from pseudo-anonymized data. A list matching participants’ personal information with their ID number will be deleted, while consent forms are maculated 10 years after study completion, after which all data will be completely anonymized. All named authors will have access to the final dataset. Pseudo-anonymized data will be entered in the *Research Electronic Data Capture* (REDCap) database, which fulfills the Danish data law for keeping patients’ records and meets GCP requirements for data management. Study personnel responsible for outcome assessments and evaluation of the findings are blinded to study medication until the data analyses are completed. Consequently, blood sample results and lists of potential adverse effects are registered in REDCap slots to which only medical doctors responsible for patient safety and the person involved in blinding of the study medication have access. Data quality is heightened by verification of data entered by outcome assessors and range restrictions on values from neuropsychological test and questionnaire scores. In addition, REDCap has a logging module which enables tracking of the entered data. As an additional precaution, registration of all primary outcome data will be double-checked by JZP.

### Participant retention

All patients are offered feedback on changes of their cognition measured with neuropsychological tests once they have completed the 3 months follow-up assessment. This will give participants insight into whether potential experiences of cognitive side effects reflect objectively verified decline or may be due to residual mood symptoms. Travel expenses with public or private transportation on days of assessments are reimbursed for all participants. Furthermore, patients will benefit from the extra care they receive from study nurses, psychologists, and medical doctors during their participation.

## Discussion

### Summary

Electroconvulsive therapy (ECT) is a safe, effective, and fast-acting treatment option for severe depressive disorders, but its use is impeded by cognitive adverse effects. Novel treatments that can counteract the ECT-associated cognitive side effects may therefore improve the current treatment strategies for depression. The present trial investigates for the first time whether short-term (four doses) add-on treatment with erythropoietin (EPO) over the first 3 weeks of ECT can counteract (or reduce) cognitive side effects of ECT and the neuronal underpinnings of such effects.

### Limitations

The extensive exclusion criteria for receiving EPO treatment are necessary to ensure patient safety. However, they limit the recruitment rate and the generalizability of findings, as the study sample will not reflect the full range of somatic and psychiatric comorbidities, treatment response, and functioning of ECT-referred MDD and BD patients in the clinic. Furthermore, concomitant pharmacological treatment may influence patients’ cognitive functioning before, during, and after study participation [49], thus potentially confounding the neuropsychological test and fMRI task data. To minimize such confounding effects, medication is kept stable for the duration of the study, if possible, and medication use is carefully recorded such that potential interaction effects with EPO can be evaluated in post hoc analyses.

### Study feasibility

Approximately 200 patients with non-psychotic unipolar disorder aged 18–60 years received ECT at the Psychiatric Center Copenhagen during a 6-year period from 2008 to 2014 [50]. In our double-blinded randomized trials of the effects of 8 weeks of EPO treatment, we included 84 patients with mood disorders over a 3-year period (2009–2012) [8, 9]. Given this and our collaboration with other hospitals within the Psychiatric Center Copenhagen, we consider recruitment of 52 patients over 28 months for the present study feasible.

### Safety procedures and monitoring

EPO is commonly used in the treatment of anemia and has a good safety profile when carefully monitored. Nevertheless, hematopoietic effects of repeated EPO administration are associated with increased risk of hypertension and blood clotting [51]. For instance, EPO has been associated with increased mortality in severely ill stroke patients with a previous history of thromboembolic disease, including patients given thrombolytic treatment [52, 53]. To ensure patient safety in this trial, we have extensive exclusion criteria to exclude participants at increased risk of thromboembolic events. There is also some concern that EPO may increase the risk of tumor growth, although the evidence for this is unclear [54]. The rare condition called pure red cell aplasia (PRCA) has been detected with subcutaneous injections and poor packaging of the EPO medicine. However, its incidence rate has fallen to 0.3/100,000 patient years [55–57]. Given thorough adherence to the exclusion criteria and the weekly safety monitoring, we evaluate the risk of such severe side effects and adverse events of EPO treatment in this trial to be extremely low. Since reticulocyte counts constitute the first indicator of PRCA, these counts will be carefully monitored. We observed no serious adverse events of 8 weekly EPO infusions in the proposed dose and administration form in our previous studies including patients with mood disorders [8, 9]. EPO-related hematocrit levels increased to an extent that necessitated bloodletting in 5 (14%) of the 35 EPO-treated patients in weeks 3–8 (only 1 in week 3), which corresponds to observations by Ehrenreich et al. [15, 16]. We discontinued EPO treatment in six patients (17%) after 5–7 weeks due to thrombocyte level increase. Since we give only 3 weeks of EPO treatment in the present study (four doses), we do not expect such side effects. Accordingly, safety monitoring involves thorough medical examinations, blood pressure measures, blood sampling, electrocardiography (ECG), and the measurement of additional safety parameters at baseline, weekly during the study, and at 3 weeks after EPO/placebo treatment completion. Patients will be informed of all potential adverse effects before randomization and told that iron supplements (which increase hematocrit levels) are prohibited. Although the risk of potential thrombosis or suspected PRCA is low, participants will be given a pocketsize plastic card with instructions about what to do and contact details to medical doctors at the local emergency department in case of these symptoms.

### Ethical considerations

Risks and disadvantages of participating in the study are minimal based on our previous EPO trials [8, 9] and the described exclusion criteria, precautions, and the established treatment plan in cases of side effects or adverse events. Indeed, the lack of effective treatments for ECT-related cognitive side effects and preliminary evidence for neurotrophic and cognitive properties of EPO underlines the benefits of being randomized to the active treatment group. Randomization to placebo for half of the patients will be disappointing compared to receiving active treatment. Nevertheless, the use of a placebo group is necessary for assessing the potential pro-cognitive efficacy of EPO. It is demanding for these very ill patients to undergo cognition and fMRI assessments. Therefore, we have limited cognition assessments to 1½ h twice during the study period and once at the 3 months follow-up, and fMRI scan assessment to 45 min once during the study period. Although the fMRI procedure is safe and non-invasive, some patients may experience claustrophobia and/or anxiety during scans. Patients may benefit from the extra care and close contact with medical doctors, psychologists, and a research nurse during their participation, which has previously revealed beneficial effects [58].

### Perspectives

If EPO is found to alleviate cognitive side effects of ECT, this could have important implications for future treatment strategies for severe depression and for the scientific understanding of the neurobiological etiology of ECT-related cognitive decline in patients treated with ECT.

## Trial status and dissemination

Participant enrollment was initiated in June 2017 and is expected to be completed by December 2019. Findings will be disseminated in peer-reviewed scientific journals and presented at scientific meetings and conferences. Author eligibility is assessed with the Vancouver Convention.

## Additional file

Additional file 1: SPIRIT 2013 checklist: recommended items to address in a clinical trial protocol and related documents. (PDF 132 kb)

## Abbreviations

AMI-SF: Columbia University Autobiographical Memory Interview-Short Form
BD: Bipolar disorder
BDI-21: Beck Depression Inventory, 21 items
BDNF: Brain-derived neurotrophic factor
BOLD: Blood-oxygen-level dependent
BT: Bitemporal
CANTAB: Cambridge Neuropsychological Test Automated Battery
COBRA: Cognitive Complaints in Bipolar Disorder Rating Assessment
CTQ: Childhood Trauma Questionnaire
ECG: Electrocardiography
ECT: Electroconvulsive therapy
EPO: Erythropoietin
fMRI: Functional magnetic resonance imaging
HDRS-17: Hamilton Depression Rating Scale (17-item version)
hsCRP: High-sensitivity C-reactive protein
ISBD: International Society for Bipolar Disorders
ITT: Intention to treat
MDD: Major depression/major depressive disorder
MINI: Mini International Neuropsychiatric Interview
PRCA: Pure red cell aplasia
RAVLT: Rey Auditory Verbal Learning Test
RBANS: Repeatable Battery for the Assessment of Neuropsychological Status
RUL: Right unilateral
RVP: Rapid Visual Processing
SPSS: Statistical Package for Social Sciences
TMT: Trail Making Test
TRD: Treatment-resistant depression
UD: Unipolar disorder
WAIS: Wechsler Adult Intelligence Scale
